# Cellular immune states in SARS-CoV-2-induced disease

**DOI:** 10.3389/fimmu.2022.1016304

**Published:** 2022-11-23

**Authors:** Keywan Mortezaee, Jamal Majidpoor

**Affiliations:** ^1^Department of Anatomy, School of Medicine, Kurdistan University of Medical Sciences, Sanandaj, Iran; ^2^Department of Anatomy, School of Medicine, Infectious Diseases Research Center, Gonabad University of Medical Sciences, Gonabad, Iran

**Keywords:** SARS-CoV-2, CD8^+^ T cell, dendritic cell (DC), regulatory T cell (Treg), myeloid-derived suppressor cell (MDSC), neutrophil, monocyte, immunity

## Abstract

The general immune state plays important roles against severe acute respiratory syndrome coronavirus 2 (SARS-CoV-2) infection. Cells of the immune system are encountering rapid changes during the acute phase of SARS-CoV-2-induced disease. Reduced fraction of functional CD8^+^ T cells, disrupted cross-talking between CD8^+^ T cells with dendritic cells (DCs), and impaired immunological T-cell memory, along with the higher presence of hyperactive neutrophils, high expansion of myeloid-derived suppressor cells (MDSCs) and non-classical monocytes, and attenuated cytotoxic capacity of natural killer (NK) cells, are all indicative of low efficient immunity against viral surge within the body. Immune state and responses from pro- or anti-inflammatory cells of the immune system to SARS-CoV-2 are discussed in this review. We also suggest some strategies to enhance the power of immune system against SARS-CoV-2-induced disease.

## Highlights

•Impaired pDCs and exhaustive T cells occur in severe SARS-CoV-2-induced disease.
•Tregs act differently in acute vs. chronic phase of the disease.
•MDSC expansion causes T-cell dysfunction in severe disease.
•Low Treg fraction causes more severe disease in adults vs. children.
•Co-adjuvant with O_2_ boosts immunity against SARS-CoV-2.

## Introduction

Severe acute respiratory syndrome coronavirus 2 (SARS-CoV-2) is a beta coronavirus that emerged first in China. SARS-CoV-2 is regarded as the etiological factor of coronavirus disease 2019 (COVID-19). The pandemic of SARS-CoV-2-induced disease is one of the current health issues causing a huge number of deaths worldwide (1). Clinical presentations show high variations in symptoms and severity of the disease (2). Patients with SARS-CoV-2-induced disease may be asymptomatic or show severe pneumonia (3). SARS-CoV-2-induced disease varies from mild to acute respiratory disease syndrome (ARDS), which is life threatening. Dysregulated immune response is behind the pathogenesis of ARDS (4).

An urgent priority is the understanding of protective responses from immune system against SARS-CoV-2 infection (1). SARS-CoV-2 induces unrestrained generation and secretion of different cytokines into the bloodstream. A consequence of this surge of inflammatory mediators is the systemic inflammation and dysregulation of responses from innate and adaptive immunity and further infiltration of different immune effector cells into various organ tissues. Overexpression of cytokines and exaggerated immune responses finally cause organ dysfunction and cytotoxicity (2). In this review, we aimed to discuss the immune state and responses from pro- or anti-inflammatory cells of the immune system to SARS-CoV-2 infection and suggest some potential strategies for strengthening the activity of immune system against this virus. Here, we have made all our effort for gathering and interpreting information about the position of immune cells in SARS-CoV-2-induced disease. We described controversies in this context and gave a reasonable answer to them. Referring also to our background knowledge over immune cells and immune state and conditions, such as hypoxia and its impact on cellular immunity in other diseases, we have made our interpretations more justifiable to the whole immune state affected by SARS-CoV-2 infection. The novelty of this study is the whole overview on cellular immune states and conditions affecting functionality of different immune cells in patients with SARS-CoV-2-induced disease.

## SARS-CoV-2: structural proteins

SARS-CoV-2 is a single-strand RNA enveloped virus with a diameter of 40–60 nm and the largest genome compared with other RNA viruses (5). SARS-CoV-2 RNA encodes four structural proteins: membrane, nucleocapsid, envelope, and spike. The structural membrane, nucleocapsid, and envelope proteins are important for genomic stability and replication of the virus, and the spike protein includes S1 and S2 subunits that allow viral fusion to the membrane of target cells and its further cellular entry (6). S1 subunit contains a receptor-binding domain (RBD) for allowing engagement of the virus with angiotensin-converting enzyme 2 (ACE2) expressed on the surface of target cells, and the S2 subunit allows further viral fusion. Nasal epithelial cells, tracheal and bronchial epithelial cells, and type II pneumocytes highly express ACE2 and are locations for initial viral infection and spread (7). The S protein of the virus is the primary target for developing vaccines (1). However, there are also vaccines developed for the full inactivation of SARS-CoV-2 (8).

## Immune cells in SARS-CoV-2-induced disease

**Figure 1:**
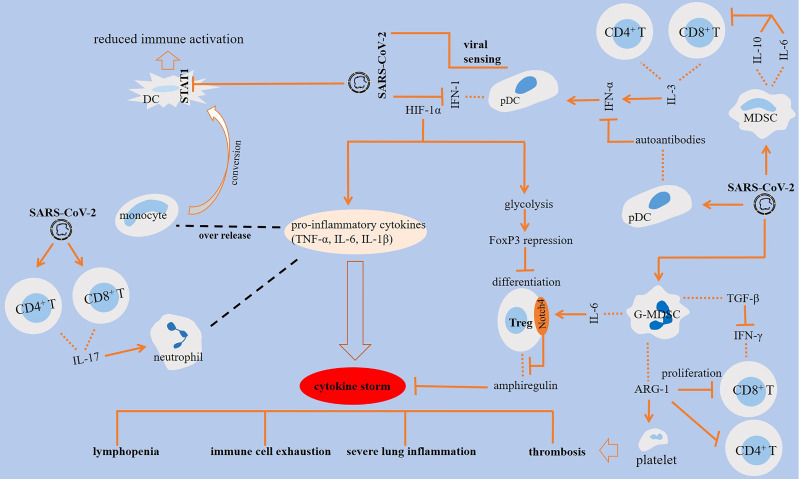
Interactions among cells of the immune system in SARS-CoV-2-induced disease. SARS-CoV-2 antagonizes STAT1 phosphorylation, which is linked to the reduced immune activation in monocyte-derived dendritic cells (DCs). The impact of SARS-CoV-2 on DCs results in the production of autoantibodies against interferon (IFN)-1. Activation of CD8^+^ T cells is essential for virus clearance, and the activity of CD4^+^ T cells is important for the activation of CD8^+^ T cells and optimal antibody responses. IL-3 released from CD4^+^ and CD8^+^ T cells stimulates IFN-α, which is further linked to the higher fraction of plasmacytoid DCs (pDCs). The effector activity of T cells is suppressed by SARS-CoV-2 inducible effect on myeloid-derived suppressor cells (MDSCs) and further release of ILs 6 and 10 from the cells. IL-6 stimulatory effect on Notch4 expression from regulatory T cells (Tregs) contributed to severe lung inflammation *via* hampering release of amphiregulin. Granulocytic (G)-MDSCs also suppress the proliferation of T cells through the release of arginase-1 (ARG-1), which contributed to lyphocytopenia. ARG-1 activity and the resultant low L-arginine level contributed to platelet activation and thrombosis. In addition, MDSCs block IFN-γ production from T cells through releasing transforming growth factor (TGF)-β.

Complex interactions are occurring in cells of the immune system upon encountering SARS-CoV-2, a summary of which is presented in **Figure 1**.

### Dendritic cells

DCs are a heterogeneous population and the most potent antigen-presenting cells (APCs) of the immune system, and their activity is important for supporting innate immunity and activation of adaptive responses from CD4^+^ and CD8^+^ T cells (7, 9). DCs present two functional subtypes: conventional (or myeloid) (cDCs) and plasmacytoid (pDCs). cDCs further include cDC1 (CD141^+^) and cDC2 (CD1c^+^) subsets (9). CD141^+^ DCs are localized in the vascular wall and mucosa and are involved in the activation of responses from T-helper 1 (Th1) cells (5). CD1c^+^ DCs support responses from CD4^+^ T cells. CD1c^+^ DCs stimulate the activity of follicular helper T (Tfh) cells, which are known as the key promoter of humoral adaptive immunity against viral infections (9). Another subset is also considered for cDCs, which is the monocyte-derived DC (mDC), also called DC3. mDCs express CD1c, CD11c, CD14, and CD163 (7). Plasmatic level of soluble CD14 and CD163 is higher in patients with SARS-CoV-2-induced disease compared with healthy donors, and their assessment is indicative of the severity of the disease (10).

pDCs are a specialized subset of DCs that display a morphology of plasma cells. The cells express human leukocyte antigen class DR (HLA-DR), CD4, CD123, and toll-like receptors (TLRs) 7 and 9 (11). pDCs are presented in lung parenchyma and airways and are involved mainly in the production of type I interferon (IFN1) (5), namely, IFN-α (12). pDCs are, in fact, considered as IFN factory in the immune system in which the production of IFN by the cells exceeds the amount generated from other cells (13), thereby displaying high anti-viral functionality (7). Three effector subsets of pDCs are P1, P2, and P3. P1 is contributed to the production of IFN-I and acts in innate immunity, P2 displays a mixed (innate and adaptive) function, and P3 stimulates T cells (14).

DCs show reduced fraction in the blood of patients with severe SARS-CoV-2-induced disease. CD123 is a marker for pDCs. In patients with severe disease, CD123^high^ pDCs are almost lost in the lung and depleted in the blood, whereas CD1c^+^ DCs migrate preferentially from the blood into the lung (15). Reduced frequencies of cDCs and pDCs in the peripheral blood of severe/critical cases and the increased fraction of these cells in convalescent individuals is reported by Chen and colleagues (16). Reduced DC fraction is thus linked to a more severe disease. Pérez-Gómez and colleagues reported that the reduced fraction of pDCs and CD1c^+^ DCs persists for 7 months after infection with SARS-CoV-2 (12).

Defective defense from APCs is occurring in severe disease. Zhou and colleagues in a study evaluated responses from T cells and DCs during the acute phase of disease. Both cell types showed functional impairment, and there was a delayed response from antigen-specific T cells (nucleocapsid protein- and RBD-specific T cells) despite the rapid generation of neutralizing antibodies (17). pDC activation into effector subsets (P1–3) is induced by SARS-CoV-2 in an initial phase compared to the late-stage severe disease (14). Saichi and colleagues in a single-cell RNA sequencing assay announced multi-process defects in blood APCs in patients with severe disease. The defects were augmented pDC apoptosis, reduced innate sensors and major histocompatibility complex (MHC)-II representation on cDC1c^+^ DCs, and downregulated genes related to the stimulation of anti-viral IFN pathway (18). SARS-CoV-2 also impairs maturation of DCs (7) and monocytes (19), thereby hampering effective activation of adaptive immunity against the virus.

IFN-I deficiency within the blood is a hallmark of the severity of SARS-CoV-2-induced disease (20). The production of IFNs is reduced in pDCs (13). A link between impaired IFN-1, delineated by lack of IFN-β and low IFN-α formation, with a severe/critical disease is identified. Deficient IFN-I activity is positively related with exacerbation of inflammation and persistent viral load (20). Impaired generation of IFN-α and the resultant low plasma level of this cytokine in patients with SARS-CoV-2-induced disease is also reported by Arunachalam and colleagues (20). In patients with a critically ill disease, systemic changes are occurring in the immune system as a result of DC-derived autoantibodies against IFN-I (21). TNF and nuclear factor kappa B (NF-kB) are mediators of hypoxia and pro-inflammatory signaling that are upregulated in pDCs (13). The activity of IL-6, TNF-α, and NF-kB is partly related to the SARS-CoV-2-related inflammation (20). IL-3 is another cytokine, its concentration of which is linked with disease severity. Patients with high viral load show low IL-3 level. IL-3 level is positively linked with increased proportion of circulating pDCs. IL-3 has been found to be related possibly with enhanced IFN-α activity, suggesting a protective role mediated by IL-3 against SARS-CoV-2-induced disease (22).

### CD8^+^ T cells

CD8^+^ T cells or cytotoxic T lymphocytes (CTLs) are powerful cells for eliminating intracellular pathogens and tumor cells (23). CD8^+^ T cells belong to the adaptive immunity, and they are the frontline defensive cells against cancer (24). Based on a study, responses from CD8^+^ T cells were found in more than 75% of convalescent patients (25), which is indicative of their important anti-viral activities. An effective defense against SARS-COV-2 is the need for immunity from both T cells and antibodies. Activation of CD8^+^ T cells is effective for virus clearance, and the activity of CD4^+^ T cells is essential for the activation of CD8^+^ T cells and optimal antibody responses. S-protein specific CD8^+^ and CD4^+^ T cells were observed in the blood of 70% and 100% of convalescent individuals, respectively (26). Early response from CD8^+^ T cells against SARS-COV-2 is protective, whereas late responses are seemingly destructive, possibly due to amplifying inflammatory responses associated with cytokine storm and lung damage (27). SARS-CoV-2-specific responses from CD4^+^ and CD8^+^ T cells soon after the onset of symptoms is linked with the fast clearance of the virus and a mild disease. A delayed response is associated with poor clinical outcomes (28). Wang and colleagues evaluated the immune state in survived and deceased patients. They described early, middle, late, and end stages for survived and deceased patients based on the time from the start of symptoms, representing ≤10, 11–20, 21–30, and >30 days, respectively. In deceased patients at early, middle, and late stages, there were hypofunctional, hyperfunctional, and anergic immune states, respectively. The proportion of CD8^+^ T cells was lower for all stages in deceased vs. survived patients (29).

SARS-CoV-2 induces a terminal differentiation state in T cells. T cells display a lower proliferation rate, an outcome of which is the reduced fraction of such important anti-viral cells (30). The fraction of CD8^+^ T cells along with T-helper memory cells is reduced in the peripheral blood of patients with severe disease, but CD4^+^-naive T cells are increased (27). During the acute phase of the disease, CD4^+^ T cells have higher fraction compared with CD8^+^ T cells, with a decrease in the latter an indicative of weak responses. Results of the study by Zhou and colleagues indicate the importance of both antibody surge and CD8^+^ T cell functionality for reducing the severity of condition in patients with SARS-CoV-2-induced disease (17). Besides their role for viral clearance, CD8^+^ T cells play an important role for generating immunological memory and thereby promoting a long-lasting immunity against viral infection (31). Patients with a history of SARS-CoV-2-induced disease will develop memory T cells for reinvigorating responses against re-entry of the virus (32). In patients recently vaccinated for SARS-CoV-2, there is a possibility of higher responses from effector memory T cells when they have had prior Tetanus–Diphtheria–pertussis (Tdap) or Measles–Mumps–Rubella (MMR) vaccination, thereby experiencing reduced disease severity (28).

### Natural killer cells

NK cells are defined as the two main subtypes: CD56^dim^ CD16^bright^ and CD56^bright^CD16^dim/neg^ (33). CD56^bright^ cells present low cytotoxic capacity. CD56^dim^ cells, by contrast, show high cytotoxic potential (34). In patients with SARS-CoV-2-induced disease, there is a severe reduction in the NK cell number (15). It was found that NK cells are reduced only in patients with severe (not mild) disease (35). NK cells are low in number but are hyper-active. The cells present a characteristic of NK^dim^ phenotype through evolving cytotoxic and pro-inflammatory roles (15).

The number of perforin-expressing NK cells was compared in intensive care unit (ICU) patients (vs. non-ICU) cases, and it was found that there was a low NK cell proportion in patients admitted at the ICU (36). Inflammatory and immunological profile of NK and T cells was also checked by Chen and colleagues in patients with SARS-CoV-2-induced disease and in convalescent individuals. In patients with severe or critically ill disease, there were high levels of inflammatory cytokines, hyperactive CD8^+^ T cells, and NK cells with reduced cytotoxic capacity (16). Such NK cells showed reduced cytotoxic activity possibly due to their hyper-active state. Elevated expression of programmed death-1 receptor (PD-1) on the surface of T cells and NK cells in patients with SARS-CoV-2-induced disease is indicative of this hyper-active state and their further exhaustion and dysfunctionality (37). PD-1 is a checkpoint receptor, in which its increased expression on the surface of T cells and NK cells in an environment with high presence of its ligand programmed death ligand 1 (PD-L1) is considered as a marker of cellular dysfunctionality (38).

### Monocytes and macrophages

Monocytes are cells of innate immunity that represent 10%–15% of peripheral blood mononuclear cells (PBMCs) (39). Monocytes are classified into classical, inflammatory transitional, and non-classical subsets. Classical monocytes are immature and are designated as CD14^++^CD16^−^ cell subset, whereas the other two subtypes are inflammatory and more differentiated cells. Transitional cells are CD14^+^CD16^+^, while non-classical cells are CD14^−^ CD16^++^ (9). Macrophages and DCs are acting as APCs for priming naive T cells and promoting their activation. Macrophages are plastic cells that have two subtypes: M1 macrophage is a classical and pro-inflammatory subtype, whereas M2 macrophage is an alternatively activated and anti-inflammatory subset (40–42). M1-like macrophages are more active in the early stages of anti-viral immune responses, while M2-like macrophages are active during late stages and act in initiating the resolution of inflammation. In fact, after induction of an inflammatory response, a shift from M1- to M2-like phenotype is effective for initiating such resolution and for recovering tissue homeostasis. Imbalanced activity of M1- to M2-like macrophages exacerbates the pathogenesis of SARS-CoV-2-induced disease (43). The dysregulation of mononuclear phagocytes is linked with disease severity (44). Monocyte-derived macrophages are known as the key promoter of inflammation in SARS-CoV-2-induced disease. By contrast, anti-inflammatory M2 macrophages contributed to the resolution of inflammation. Such cells regulate the duration and magnitude of acute inflammation through releasing protectins, resolvins, and maresins (45).

The fraction of non-classical monocytes is increased in the peripheral blood and within the lungs of patients with SARS-CoV-2-induced disease, particularly in cases with more severe disease. To explain, Tincati and colleagues evaluated the immune state in two categories of patients: mild SARS-CoV-2-induced disease with PaO_2_/FiO_2_ > 300 and severe disease with PaO_2_/FiO_2_ < 200. They noticed the higher fraction of circulatory non-classical monocytes along with increased polarization toward Th1 cells with pro-cytolytic activity in the severe vs. mild disease (46). Sánchez-Cerrillo and colleagues found an enrichment of lungs with inflammatory transitional and non-classical cells in patients who were developing ARDS. It was found that there was more preferential infiltration of inflammatory monocytes compared with DCs during the progressive stage of SARS-CoV-2-induced disease (9). A work by another group showed a link between CD169 expression on monocytes with their enhanced capacity to stimulate CD8^+^ T cells. CD169^+^ monocytes were detected in CD14^+^ cells (classical and intermediate [transitional] monocytes) and observed in bronchoalveolar fluid and blood of patients with SARS-CoV-2-induced disease. These CD14^+^ CD169^+^ cells were, thus, considered as a promising source in vaccination therapy (39).

**Figure 2:**
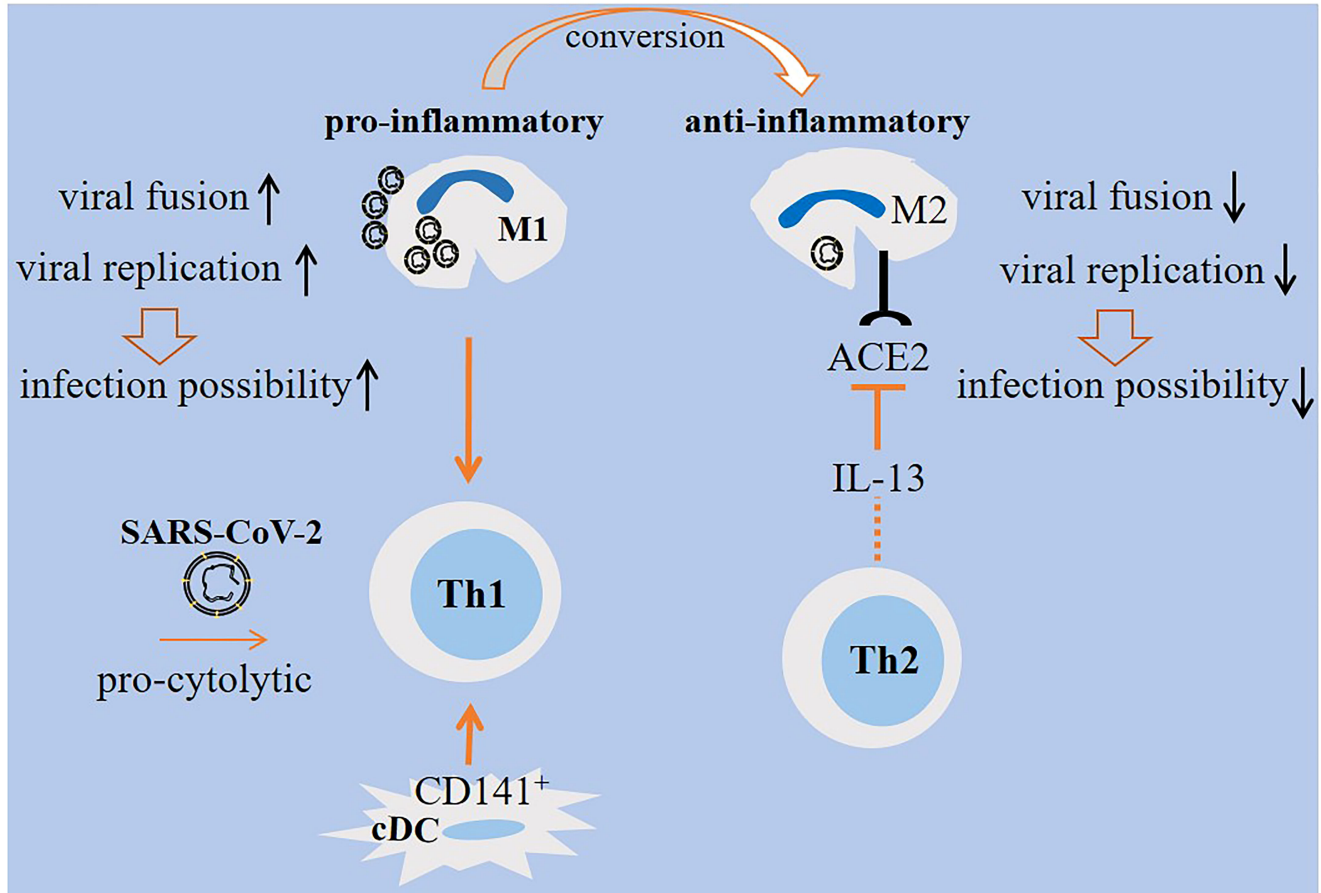
M1/M2 cellular states in SARS-CoV-2-induced disease. M1 macrophages are pro-inflammatory and are active during the early stage of viral entry, whereas M2 macrophages are anti-inflammatory that are active during late stages. Viral replication and fusion are high in M1 macrophages, whereas the rate of replication is low in M2 macrophages. Lower lysosomal pH favors more viral degradation in lysosomes of M2 macrophages. Therefore, M1 macrophages are more prone to viral load and lung infection. The expression of angiotensin-converting enzyme 2 (ACE2) on M2 macrophages is reduced by IL-13 released from T helper 2 (Th2) cells, which is indicative of a level of resistance among M2 macrophages. Th1 cells are induced by M1 macrophages. Th1 polarity with pro-cytolytic activity is also induced by severe SARS-CoV-2. A shift from M1- to M2-like phenotype is required for promoting resolution of inflammation.

Monocyte-derived macrophages are permissive to SARS-CoV-2 and promote host cell death. To explain, Yang and colleagues evaluated the interplay between SARS-CoV-2 with mDCs and macrophages. Both cell types were permissive to the virus, but did not allow viral replication. Reduced IFN response occurred in both cells, but the expression of pro-inflammatory cytokines/chemokines was triggered only in monocyte-derived macrophages (47). Zheng and colleagues announced that SARS-CoV-2, although showing abortive infection and no efficient replication in monocyte-derived macrophages, induced profound death of host cells (48). A report by another group showed no possible link between macrophages and mDCs with first wave of cytokine storm in patients with SARS-CoV-2 in which none of the infected cells generated pro-inflammatory cytokines*. In vitro* assays showed the sensitivity of such cells to the SARS-CoV-2 infection (49). SARS-CoV-2 inhibitory effect on STAT1 phosphorylation contributed to the reduced immune activation in mDCs (47). A study by Lv and colleagues delineated diverse responses from M1/M2 alveolar macrophages upon exposure to SARS-CoV-2. M1 macrophages were more prone to the viral fusion and replication due to having lower endosomal pH. By contrast, M2 macrophages were able to deliver the virus toward lysosomes for further degradation due to their lower lysosomal but higher endosomal pH (50) (**Figure 2**).

M2 macrophages expressing PD-L1 are protected against severe SARS-CoV-2-induced disease. Trombetta and colleagues evaluated a link between myeloid cell immune activation and phenotype with recovery from SARS-CoV-2-induced disease. PD-L1^+^ M2-like monocytes had the highest proportion among ICU cases at discharge. The cells with such high fraction represented a phenotype of classical monocytes (CD14^++^CD16^−^). The percentage of PD-L1^+^ M2-like monocytes was inversely linked with levels of chemokines and inflammatory cytokines related to the IFN pathway and was linked directly to the anti-SARS-CoV-2 Ig (IgG and IgM) titer. A marked decrease in the proportions of CD141^+^ mDCs and pDCs was also identified in all cases, which were partially recovered in non-ICU patients at discharge (51).

### Neutrophils

Neutrophils comprise over 50% of human leukocytes in the peripheral blood and are known as the most frequent effector cells of innate immunity (52). High fraction of neutrophils and increased neutrophil-to-lymphocyte ratio are considered as initial sign of SARS-CoV-2-induced disease (53). SARS-CoV-2 induces neutrophil infiltration and stimulates a hyperactive state in such cells for promoting hyperinflammation. Hammoudeh and colleagues evaluated multi-organ involvement in SARS-CoV-2-related cytokine storm. Results showed dysregulation in cytokine generation by inflamed pulmonary and extra-pulmonary organs, such as the kidney, liver, and heart. It was also found that there was upregulation of genes related to the neutrophil-associated immune responses mainly in the lung tissue. By contrast, B cells, T cells, DCs, and monocytes showed almost similar dysregulated responses in all types of tissues (2). Vanderbeke and colleagues investigated the immune cell basis of SARS-CoV-2-related hyperinflammation and severity. A fundamental role was identified for classical monocytes and hyperactivated neutrophils in hypercytokinemia. Neutrophils showed higher fraction in the lungs of patients with severe disease. Hyperactive neutrophils were marked by the upregulation of C-X-C motif chemokine ligand 8 (CXCL8), IL-1β, and S100A12 (54). SARS-CoV-2-infected cells release CXCL2 and CXCL8 for attracting infiltration of neutrophil-inducing immune cells (6). Neutrophils are activated in response to IL-17 release from CD4^+^ and CD8^+^ T cells in SARS-CoV-2-related pneumonia. The activity of this cytokine is linked positively with strengthened inflammatory events, so it can be a target for therapy (30). The hyperactive neutrophils seemingly have an immature phenotype. In fact, hyperinflammation related to SARS-CoV-2 promotes a shift in the neutrophil phenotype toward an immature state (19).

### Regulatory T cells

Tregs are the key immunosuppressive cells (55) that migrate into areas of inflammation and contribute to the suppression of inflammatory responses (56). Tregs act for the stimulation of immune tolerance, and their presence is important for preventing inflammatory and autoimmune diseases (57). Thus, CD4^+^FoxP3^+^ Tregs play important roles for maintaining immune homeostasis (3). The reduction or defective Treg functionality is partly contributed to the excessive systemic inflammation and to the impaired lung repair in SARS-CoV-2-induced disease (56).

Tregs possibly contributed to the amelioration of anti-viral defense at early stage of SARS-CoV-2-induced disease, whereas the activity of these cells will help attenuate inflammation-induced organ damage at late stage of the disease (3). The number of Tregs is reduced in patients with a severe disease compared with mild disease cases (58), which may be a reason for immune hyperactivation and lung damage (57). A point, however, is that Treg activity can promote immunosuppression in favor of disease progression despite the counter-effect of these cells on SARS-CoV-2-related inflammatory responses. Based on a report, viral particles can be observed at 70 days after the initial diagnosis of SARS-CoV-2-induced disease, causing long duration of infection with the virus (59). Yang and colleagues in a study evaluated the immune state in patients with long vs. short duration of SARS-CoV-2 infection. In patients with long duration of disease, there was an increase in the number of Tregs, but NK cell population was decreased. NK cells were less activated in long duration compared with the short duration of disease. Therefore, immunosuppression can be a reason for promoting SARS-CoV-2 persistence (60), thereby causing chronic viral infection and high loads of antigens within the body (61).

**Figure 3:**
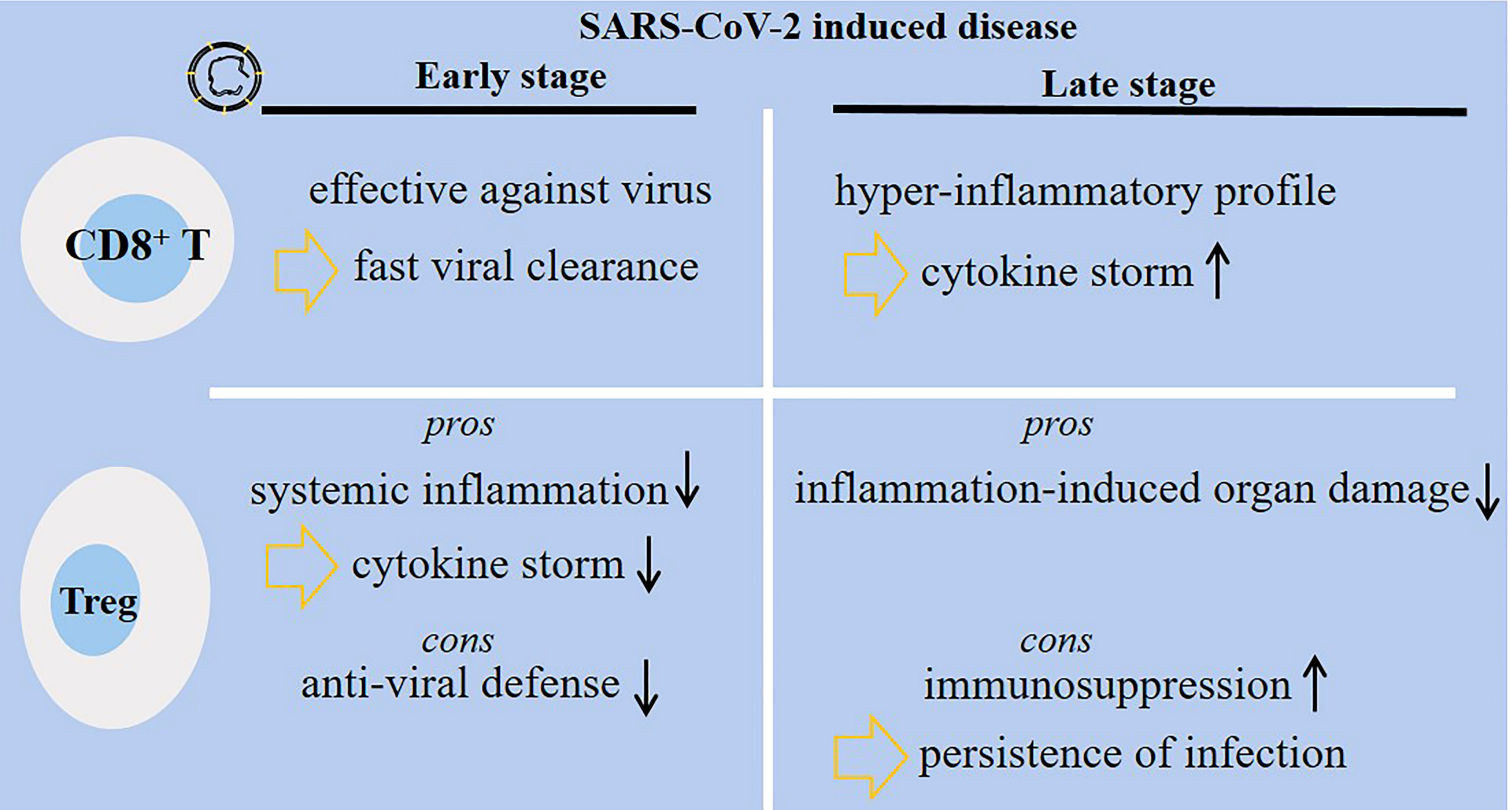
Diverse functionality of CD8^+^ T cells and regulatory T cells (Tregs) in patients with SARS-CoV-2-induced disease. During the early-stage of the disease, the activity of CD8^+^ T cells is protective, whereas the cells are hyper-active and amplify inflammatory responses associated with cytokine storm and lung damage at late stage of the disease. Tregs have their own pros and cons during each disease stage. At early stage, Tregs dampen systemic inflammation, but they can hamper anti-viral defense systems. At late stage of the disease, Tregs protect body organs from inflammatory-related damages, but they may promote immunosuppression and further persistence and delayed clearance of the virus.

Wei et al. compared the immune state in two groups of patients with SARS-CoV-2-induced disease: good (n = 3) vs. poor prognosis group (n = 3). In patients with poor prognosis at the time of exacerbation, Th2 cells and Tregs were relatively higher compared with the good prognosis group (62). However, injection of allogenic Tregs for two critically ill patients admitted at Johns Hopkins University hospital presented clinical benefits and attenuated inflammatory markers. Both patients receiving such therapy survived and were discharged from the hospital (63). To support this, Meckiff and colleagues analyzed the frequency of cytotoxic and regulatory CD4^+^ T cells in hospitalized and non-hospitalized patients. Hospitalized patients had low fraction of Tregs but high proportion of CD4^+^ cytotoxic T-follicular and T-helper cells (64). From the results, it can be postulated that there is a positive link between low Treg fraction with more severe disease. Thus, recovering the functionality and fraction of Tregs can be an effective strategy for reducing disease burden. Two points, however, require attention from what was discussed above: (1) Treg activation at early-stage of SARS-CoV-2-induced disease dampens anti-viral defense systems, and (2) Treg activation at late stage of the disease promotes immunosuppression and may cause persistence of viral infection and delayed viral clearance. **Figure 3** shows diverse functionality among CD8^+^ T cells and Tregs in patients with early- or late-stage SARS-CoV-2-induced disease.

### Myeloid-derived suppressor cells

Myeloid-derived suppressor cells (MDSCs) are a heterogeneous population of immature cells that belong to the innate immunity and take immunosuppressive activities (65, 66). MDSCs are mainly derived from granulocytes (G-MDSCs) and monocytes (M-MDSCs). M-MDSCs are CD14^+^, while G-MDSCs are CD15^+^, both of which are either negative for HLA-DR or show low expression of this factor (65). Severe SARS-CoV-2-induced disease coincides with the presence of MDSCs (44). In fact, the expansion of myeloid cell compartment is considered as a hallmark of a severe disease (67). A study by Reizine and colleagues showed that in patients with a severe disease (developing ARDS), both G-MDSCs and M-MDSCs were increased compared to the cases with moderate pneumonia, in which the fraction of both MDSC subtypes was high at day 7 after ICU admission only in patients developing ARDS (68). In another study, a huge expansion of MDSCs (90% of circulatory mononuclear cells) was identified in severe (vs. mild) disease (69). Xue and colleagues found a positive link between CD14^+^HLA-DR^low/neg^ MDSCs (M-MDSCs) with viral load in the oropharyngeal area and with the length of patient stay in the hospital (70). A high fraction of MDSCs was found within the blood but not in endotracheal or nasopharyngeal aspirates of patients with severe disease (71).

High MDSC fraction is linked negatively with T-cell functionality. The higher expansion of M-MDSCs contributed to the dysfunctional T cells in patients with severe SARS-CoV-2-induced disease. M-MDSCs release IL-6 and IL-10 to augment the rate of inflammation and accumulating Tregs and regulatory B cell (Bregs) (72). Tregs accumulated within the lung display high IL-6-induced Notch4 expression, which hampers the release of amphiregulin, a cytokine related to tissue repair, thereby promoting severe lung inflammation and a more severe SARS-CoV-2-induced disease (73). A positive relation between MDSC expansion with increased serum level of transforming growth factor (TGF)-β is reported. At the convalescent phase of SARS-CoV-2-induced disease, a reduced fraction of MDSCs is related with lower TGF-β levels (69). TGF-β is a multi-tasking and a potent pro-fibrotic cytokine with immunosuppressive activities (74–76). In patients with severe disease, TGF-β released from G-MDSCs contributed to the suppression of IFN-γ generation from T cells, which further hampers the effective elimination of the virus (77). From what was discussed above, MDSC expansion, involving both M-MDSC and G-MDSC subsets, occurs in severe SARS-CoV-2-induced disease, and the high fraction of these cells contributed to T-cell dysfunctionality. A contrast to these findings is a study carried out by Takano and colleagues evaluating MDSC subsets in Japanese patients. Here, they noticed a transient (rather than an immense) expansion of only G-MDSCs (no other subsets) in cases with severe SARS-CoV-2-induced disease. They showed that this delay or transient aggregation is beneficial in improving clinical outcomes, delineated by attenuated SARS-CoV-2-related severe lung inflammation. Such transient expansion was not seen in patients who died from the disease (67). From this study, it could be postulated that a rate of MDSCs is beneficial for patients with SARS-CoV-2-induced disease, but a high fraction of such cells will hamper T-cell functionality against this disease. To explain, lectin-type oxidized LDL receptor 1 (LOX-1) expression by MDSCs is indicative of their potent immunosuppressive activity. Coudereau and colleagues noticed an elevation of LOX-1^+^ MDSCs in patients developing ARDS, which hampers effective resolution of infection (78).

### Other cells

Mast cells are cells of innate immunity that take pathogenic roles in several inflammatory diseases. The activation of mast cells is associated with the SARS-CoV-2-related inflammatory events. Mast cells are among the fast-responder cells at the time of pathogen invasion. In the serum and lungs of patients with SARS-CoV-2-induced disease, there is a rise in the mast-cell-derived proteases (79). Mast cells are induced by chemokines synthesized during SARS-CoV-2 infection (80). Mast cell activation by the virus increases the risk of lung inflammation and fibrosis (81). Protease production by mast cells is a feature of hyperinflammation. Selective inhibition of mast cells using anti-Siglec-8 antibody is reported to suppress airway infiltration and disease severity in a model of respiratory viral infection, which is indicative of the importance of anti-Siglec-8 antibody in the attenuation of excess inflammatory events occurring during viral infection (79). However, a study by Valent and colleagues showed that in patients with mast cell activation disease, there was no elevation in the overall risk of developing toward SARS-CoV-2-induced disease, but they recommended to manage mastocytosis when complications, such as acute anaphylaxis, occur (82).

Basophil count is reduced in patients with acute disease and is correlated with poor prognosis (83). Based on a report by Rodriguez and colleagues, the proportion of the granulocyte subsets eosinophils and basophils is increased in patients passing from the acute to recovery phase, both of which were among the cells with the most dynamic changes during the severe disease. The number of basophils was also associated considerably with the production of IgG antibody from B cells against SARS-CoV-2. This is indicative of the key contribution taken by basophils to the immunopathology of SARS-CoV-2-induced disease and anti-viral defenses (84).

Eosinophils are a part of host immune defense against respiratory viruses (85). There is a report of a link between eosinopenia with acute respiratory deterioration upon SARS-CoV-2 infection and that eosinopenia may serve as a poor prognostic indicator and a marker of a more severe disease (86). Vieyra and coworkers announced that the presence of eosinophils is seemingly effective for controlling neutrophil-induced exacerbation of inflammation. They found a negative correlation between eosinophil count with neutrophil count and neutrophil-to-lymphocyte ratio. Another finding of this study was a decrease in the number of eosinophils in deceased (vs. recovered) patients (eosinophil level < 0.01 × 109/L: eosinopenia), while a rapid increase in their number was identified in recovered individuals (87). Another study demonstrated the positive link between higher eosinophil count with protection against poor outcomes from SARS-CoV-2-induced disease in patients receiving inhaled corticosteroid therapy (but not in cases without corticosteroid prescription), which is suggestive of the protective role of corticosteroids against SARS-CoV-2 infection (88). A study by Pala and colleagues showed the advantage of eosinophil involvement during the initial phase of SARS-CoV-2 infection, but eosinophilic activity in patients with severe SARS-CoV-2-induced disease is contributed to the mal-adaptive immune responses and is responsible for the immunopathology of the disease. The authors suggested the inhibition of eosinophile activation in severe SARS-CoV-2 patients as a possible strategy for contrasting harmful immunity (85). Lung eosinophilic immunopathology occurring secondary to the SARS-CoV-2 vaccination is called vaccine-associated enhanced respiratory disease. In order to reduce the risk of such immunopathology related to the eosinophilic activity, Hemmi and colleagues recommended intranasal vaccination therapy against S protein of the virus along with the application of the TLR9 agonist ODN2006 (89).

## Immune-cell-based interactions for promotion of lymphopenia in SARS-CoV-2-induced disease

G-MDSCs contributed to the impaired proliferation of T cells in SARS-CoV-2-induced disease (90). A study by Reizine and colleagues attested a positive relation between high MDSC fraction with reduced T-cell count (lymphopenia). High arginase-1 activity of MDSCs is responsible for reduced T-cell proliferation (68). Patients with SARS-CoV-2-induced disease have high fraction of arginase-1^+^ neutrophils with an immature phenotypical state (91). This is presumably indicative of the implication G-MDSCs for hampering T-cell proliferation. Xiang and colleagues found a relation between inflammation and lymphopenia with decimation of secondary lymphoid organs including spleen and lymph nodes. Here, the high release of IL-1β and IL-6 from infected DCs was found as a possible reason for the decimation of such lymphoid organs and the subsequent lymphopenia (92). High IL-6 is also released from MHC-II^low^ monocytes (13). T cells in the acute phase of disease display a unique metabolic profile. The cells present mitochondrial apoptosis that, in turn, promotes lymphopenia in this phase of SARS-CoV-2-induced disease (93).

## Angiotensin-converting enzyme 2 and immune state in SARS-CoV-2-induced disease

ACE2, which serves as the receptor for S protein of SARS-CoV-2, is expressed by DCs and macrophages. This may indicate that the cells, although contributed to the anti-viral defensive system, may act to enable viral anchoring inside the lung parenchyma (94). IL-13 reduces expression of ACE2 by M2 macrophages, which may be indicative of viral resistance of this macrophage subtype (45). In addition, as mentioned above, due to having lower lysosomal pH, the cells tend to deliver SARS-CoV-2 virus to lysosomes for further degradation, thereby hampering further replication of the virus.

Patients with active SARS-CoV-2 infection show reduced ACE2 levels due to its cellular internalization. Engagement between SARS-CoV-2 with ACE2 will lead to the internalization of ACE2 and inhibition of its activity. Thus, activation of ACE2 receptor can be a possible approach for blocking the development of the condition toward cytokine storm in patients with SARS-CoV-2-induced disease. In a study, macrophages were treated with the ACE2 receptor activator diminazene aceturate, and there was an inhibition of SARS-CoV-2-related pro-inflammatory profile by this agent. Therefore, ACE2 receptor activator can be suggested as a treatment schedule for SARS-CoV-2-induced disease (95). Diminazene aceturate can also induce vascular repair and improve alveolar remodeling. ACE2 activators can, thus, be used as an approach for controlling the escalation of the disease (96).

## Immune cells and thromboembolic events in SARS-CoV-2-induced disease

Neutrophils are strongly associated with platelets in patients with severe SARS-CoV-2-induced disease (97). Acute viral infection is linked positively with dysregulated formation of neutrophil extracellular traps (NETs) (52). Excessive generation (52) and release (54) of NETs from neutrophils is related positively with severe disease. NETs restrict viral spreading, but they can promote thrombi formation through engaging platelets (6). Platelet activation is also increased when L-arginine concentration is low (98). L-Arginine is degraded by arginase-1, and G-MDSC is a cell type displaying arginase-1 activity (65). High levels of arginase is produced from G-MDSCs in severe SARS-CoV-2-induced disease (99). Therefore, G-MDSCs are linked positively with the development of thromboembolic events through their high arginase activity.

## Hypoxia and cellular immunity in SARS-CoV-2-induced disease

Severe SARS-CoV-2 infection reduces O_2_ levels within the blood and affected tissues (100). Hypoxia-inducible factor (HIF)-1α is a key mediator of severe or acute hypoxia (101). SARS-CoV-2 activates HIF-1α, the activity of which exacerbates generation of pro-inflammatory signaling and augments the rate of cytokine storm (102). Monocytes are more recruited toward hypoxic tissues and differentiated into macrophages (100). SARS-CoV-2 infection promotes hypoxemia, which further causes expression of HIFs by immune cells (103). The activity of HIFs on macrophages promotes their local aggregation and activation (103). The promoter of *FoxP3* gene contains an HIF-responsive element (104). FoxP3 shows repressed expression in lungs of patients with severe SARS-CoV-2-induced disease. HIF-1α promotes aerobic glycolysis, which is further contributed to degradation of FoxP3, thereby blocking differentiation of Tregs (105).

Designing a strong vaccination system that is effective in generating sufficient levels of neutralizing antibodies after incubation with one dose is a preferred approach concerning the shortage of vaccine for SARS-CoV-2 virus. A strategy that can boost the rate and binding activity of antibody titers is to use O_2_ as a co-adjuvant with SARS-CoV-2 vaccination. O_2_ has strong immunological activity, and its co-adjuvant is an effective approach for potentiating vaccine potency. O_2_-generating COVID-19 cryogel-based vaccine (O_2_-Cryogel_VAX_) is an example in this context. The application of O_2_-Cryogel_VAX_ in mice has found to induce high titers of antibodies and stimulate their potent neutralizing activity. Sustained generation of neutralizing antibodies along with promotion of local recruitment of immune cells is a virtue for the use of O_2_-Cryogel_VAX_ (106).

## Immune state and SARS-CoV-2-induced disease in children vs. adults and elderly

Diverse susceptibility among children and elderly individuals to SARS-CoV-2 infection is explained in the context of the immune system. The immunological profile in response to SARS-CoV-2 is different in children compared with adults. In children, immunophenotype is less inflammatory, and the humoral immunity is as strong as what is seen in adults (107). Based on the results of one study, there is increased proportion of CD63^+^ neutrophils in children but no response from these cells in adults during the acute phase of SARS-CoV-2-induced disease, and the impact of CD63 on neutrophil activation and secretion of pro-inflammatory mediators are all indicative of diverse responses from innate immunity between children and adults (108). Neeland and coworkers evaluated the immunological mechanism behind milder symptoms in children compared to adults. Children during acute phase of the disease had decreased proportions of all subsets of circulating monocytes including classical, transitional, and non-classical subtypes, whereas only the non-classical subtype of monocytes showed reduced fraction in adults (108). Elderly individuals, by contrast, have increased number of inflammatory transitional monocytes, which is a reason for more vulnerability to SARS-CoV-2-induced disease among aged individuals (109). Aged individuals present loss of Treg functionality, which results in the difficulty in controlling immune responses, thereby enhancing the inflammation rate and inflammatory storm upon encountering SARS-CoV-2. Thymus is an organ that acts in mediating adaptive immune responses and immunomodulation. Tregs are active during the early life period and take function as immunomodulatory cells. T cells destroyed by virus are replaced in the thymus, so shrinkage of this organ during adolescence period may be one possible reason for more severe SARS-CoV-2-induced disease in adults compared with children (110).

## From therapeutic standpoint

Induction of antigen-specific CD8^+^ T cell immunity provides appropriate protection against SARS-CoV-2. A strategy to achieve this aim is to use DNA vectors expressing different proteins of SARS-CoV-2. This approach is effective through eliciting CD8^+^ T-cell responses against various antigens in a single injection (111). N^361-369^-specific T-cell receptors (TCRs) can be obtained from individuals recovered from SARS-CoV-2-induced disease and can be exploited as a useful strategy for promoting CD8^+^ T-cell cytotoxicity and effector cytokine release from CD4^+^ T cells. Activation of CD8^+^ T cells and their cytolytic activity by the T-cell epitope N^361–369^ can cause lysis of SARS-CoV-2-infected cells and promote viral clearance in patients with SARS-CoV-2-induced disease (25).

Lipid-based nanoparticle vaccine platform (NVP) can be developed to promote long-lasting immunity against SARS-CoV-2. NVPs are loaded with SARS-CoV-2 antigens for inducing antigen-specific antibodies. Such NVPs are taken up by DCs and contribute to the maturation of DCs and strengthening of their antigen presentation capacity (112).

Extracellular vesicles (EVs) including exosomes can be used as a tool for developing vaccines against viral antigens. Exosomes loaded with therapeutic cargo can be used for such purpose. Exosomes interact with different cells of the immune system including NK cells, T cells, DCs, and macrophages to allow cell–cell communications and modulate anti-viral immune responses against SARS-CoV-2 (113). The reaction from CD8^+^ T cells can be induced using EVs engineered for representing viral antigens. In this way, EVs are acting as an APC. A virtue of EV-based therapy is the safety of this approach for designing virus-free vaccines due to evolving low baseline immunological profile (114).

## Conclusions

**Figure 4:**
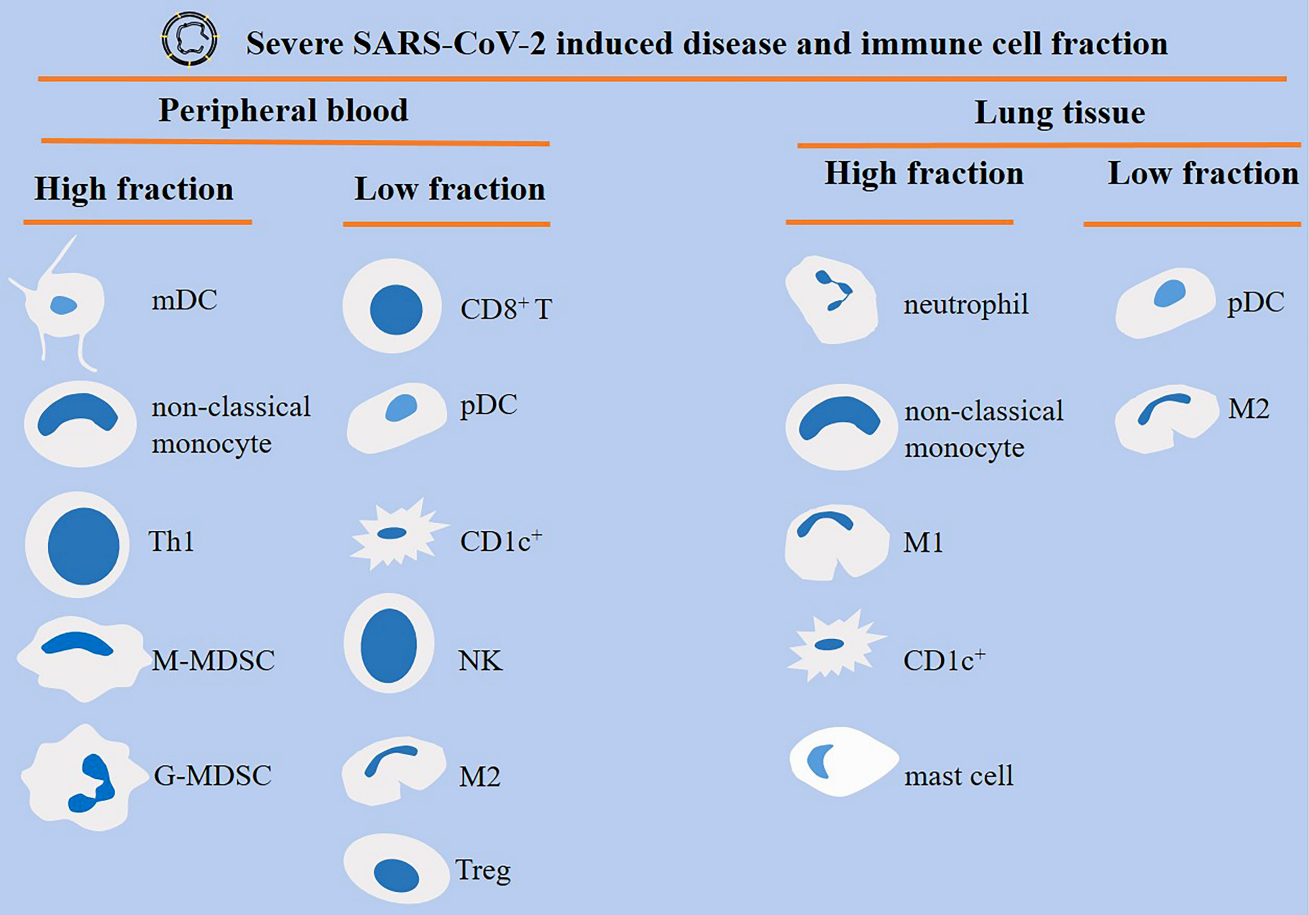
Different orchestration of cells of the immune system within the blood or lung of patients with severe SARS-CoV-2-induced disease. Monocyte-derived DCs (mDCs), non-classical monocytes, T helper 1 (Th1) cells, and myeloid-derived suppressor cells (MDSCs) are high in the peripheral blood of patients with severe disease. By contrast, CD8^+^ T cells, plasmacytoid DCs (pDCs), CD1c^+^ DCs, natural killer (NK) cells, macrophage type 2 (M2) cells, and regulatory T cells (Tregs) show low fraction within the circulation. M1 macrophages are high, whereas M2 macrophages are low within the lung of severe cases.

**Figure 5:**
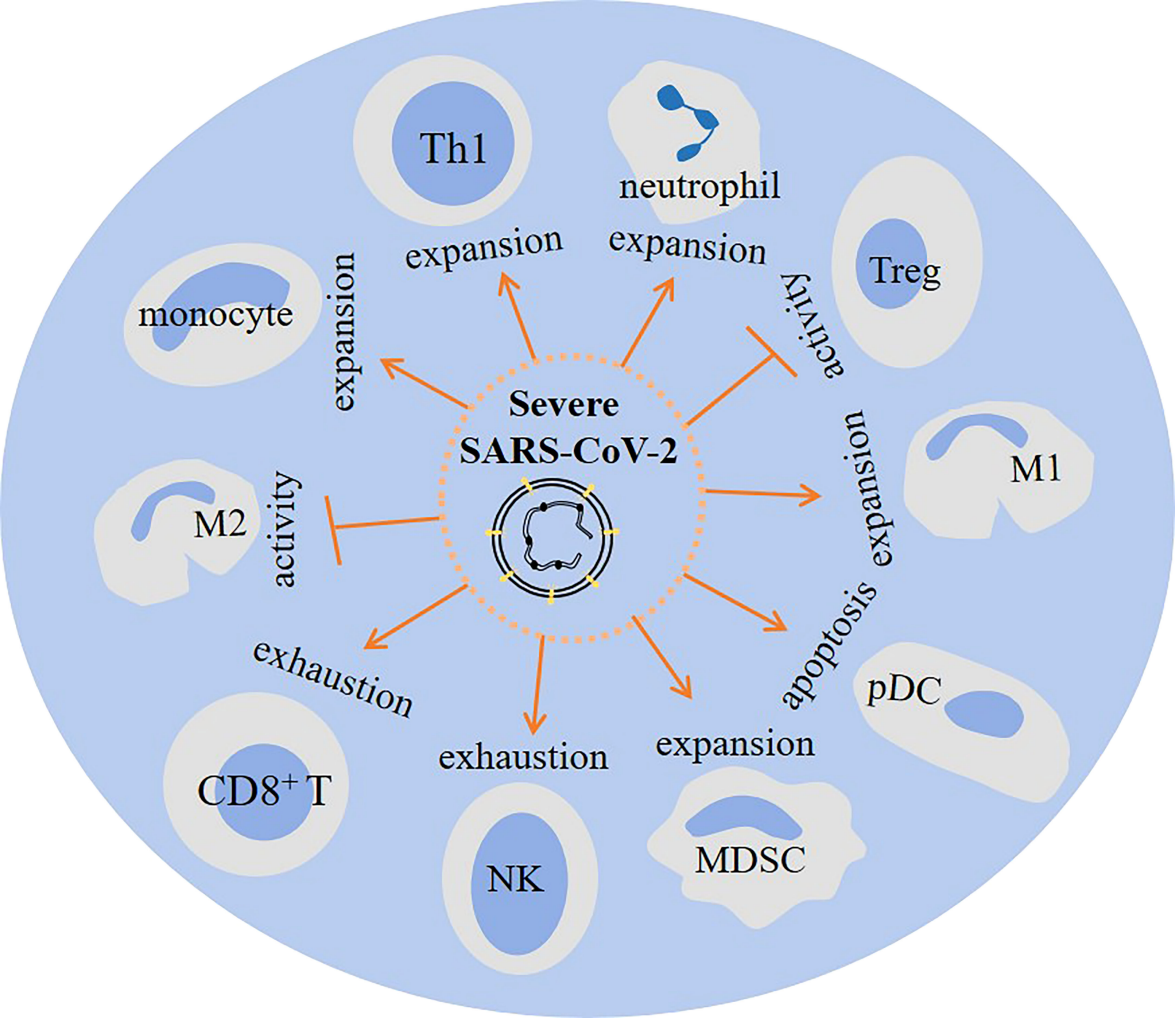
The impact of severe SARS-Cov2 induced disease on cellular immunity. In patients with a severe disease the activity of regulatory T cell (Tregs) and M2 macrophage is hampered. Whereas M1 macrophage, myeloid-derived suppressor cells (MDSCs), monocytes, neutrophils and Th1 cells show increased expansion. Other consequences of a severe infection are plasmacytoid dendritic cell (pDC) apoptosis, and CD8+ T cell and natural killer (NK) cell exhaustion.

Patients with severe SARS-CoV-2-induced disease show a dysregulated orchestration and functionality in cells of the immune system, which results in aggravation of the condition and promotion of systemic inflammation and multi-organ injury. MDSCs, neutrophils, and monocytes are highly present, whereas CD8^+^ T cells and NK cells are reduced in severe diseases (**Figure 4**). This is indicative of an immunosuppressive profile in the immune system, as depicted in the **Figure 5**. Strategies to bring back the normalization in the cellular immune state will be a valuable tool for powering the activity of the immune system against viral entry and strengthening the efficacy of vaccination therapy. Such strategy can reduce the involvement of other organs and the time of hospital stay in cases with a severe disease.

## Author contributions

Collection and revision of information, KM and JM. Conceptualization, KM. Writing, original draft preparation, review, and editing, JM and KM. Two authors have read and agreed to publish of the manuscript.

## Conflict of interest

The authors declare that the research was conducted in the absence of any commercial or financial relationships that could be construed as a potential conflict of interest.

## Publisher’s note

All claims expressed in this article are solely those of the authors and do not necessarily represent those of their affiliated organizations, or those of the publisher, the editors and the reviewers. Any product that may be evaluated in this article, or claim that may be made by its manufacturer, is not guaranteed or endorsed by the publisher.
